# A Closer Look at Aplasia Cutis Congenita: Understanding a Unique Case

**DOI:** 10.7759/cureus.61516

**Published:** 2024-06-02

**Authors:** Ala' Jumei’an, Hamza Ababneh, Mahmoud Jaradat, Ahmad Omari, Mohammad Aljbour, Mutaz Aljader, Reham Albadaineh, Gaith Alsaket, Mohammad Al Bdour

**Affiliations:** 1 Plastic and Reconstructive Surgery, Royal Medical Services, Amman, JOR; 2 Plastic and Reconstructive Surgery, Jordan University Hospital, Amman, JOR

**Keywords:** split thickness skin graft, scalp graft, congenital disease, cutis aplasia, aplasia cutis

## Abstract

Aplasia cutis congenita (ACC) is a rare congenital disease defined by the absence of skin, most commonly on the scalp. While the exact incidence remains uncertain, ACC presents a significant challenge in clinical management due to its variable presentation and associated complications. We present the case of a newborn male with a large scalp defect attributed to ACC, complicated by a life-threatening scalp hemorrhage. Despite challenges in management, including recurrent infections and failed skin grafts, the patient ultimately achieved satisfactory healing following a series of surgical interventions, including local transposition flap procedures. This case underscores the importance of a multidisciplinary approach to managing ACC, tailored to individual patient characteristics and associated risks. While discrete lesions of ACC typically have a favorable prognosis, extensive defects pose significant risks of morbidity and mortality, highlighting the need for careful consideration of treatment options and close clinical monitoring of affected individuals.

## Introduction

Aplasia cutis congenita (ACC) represents a diverse range of rare congenital disorders marked by the partial or complete absence of skin, which can manifest locally or extensively [1,2]. While ACC can occur on any part of the body, it predominantly affects the scalp. The precise incidence remains uncertain but is roughly estimated to be 0.5 to three cases per 10,000 live births [3,4]. Aplasia cutis congenita commonly presents as an isolated condition; however, it may also occur with various genetic syndromes and congenital anomalies. Although most instances of ACC arise sporadically, familial occurrences have been documented [5,6]. The exact underlying mechanisms driving the development of ACC remain unclear, with several theories proposed. In 1986, a classification system gained widespread acceptance; it delineated ACC based on factors such as the mode of genetic inheritance, site and pattern of skin defect, and the presence of related abnormalities [7].

The clinical presentation of ACC at birth can vary significantly. Diagnosis is primarily clinical, often relying on the identification of a characteristic skin defect, typically located at the vertex of the scalp. A thorough, complete physical examination is crucial to detecting any accompanying anomalies and determining the appropriate workup. Due to the wide spectrum of presentation and severity in ACC cases, treatment approaches need to be personalized. Factors such as the size and depth of the defect, the risk of complications, and clinician expertise should be taken into account [1]. Treatment could be conservative, surgical, or a combination of both, depending on the presentation of the case and the factors aforementioned [2]. 

In this report, we present a case involving a newborn diagnosed with ACC affecting both the scalp and cranium. The infant experienced a life-threatening hemorrhage, which was controlled. The defect was subsequently addressed through surgical intervention utilizing a split-thickness skin graft (STSG).

## Case presentation

The patient was a 2800 g male with a scalp defect found incidentally upon delivery. He is the first-born child of non-consanguineous parents, delivered through a cesarean section due to an inadequate pelvis and postdate pregnancy that led to fetal distress. The mother is a 39-year-old non-smoker, non-alcoholic, medically and surgically free female who had an uncomplicated pregnancy except for transient polyhydramnios in the sixth month of pregnancy, which resolved after cutting down on sugar. She didn’t experience gestational diabetes mellitus (GDM) or gestational hypertension (GHTN) or have any infections, trauma, or placental abnormalities during pregnancy.

**Figure 1:**
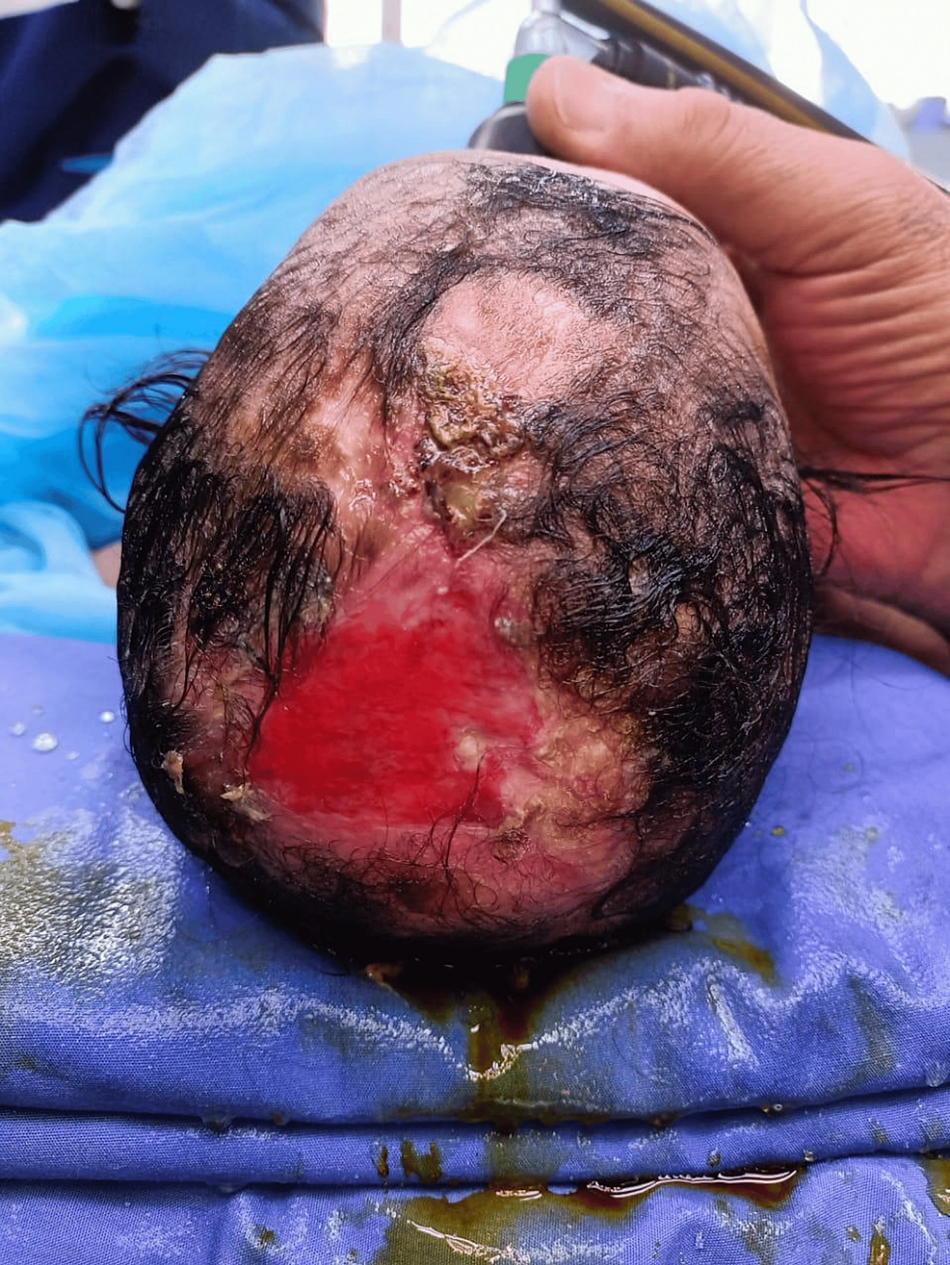
Scalp and cranium defect at seven weeks old

At birth, the newborn underwent a full physical examination, revealing no abnormalities except for the scalp defect. The newborn was admitted to the NICU for one week due to life-threatening bleeding from the aplasia cutis, during which the bleeding was managed by direct pressure with gauze. Following discharge, the newborn received daily local antimicrobial ointment for one week at home but was readmitted to the hospital for three weeks due to recurrent bleeding from the same scalp defect. The patient was referred to the plastic surgery department due to persistent bleeding complications. The defect was large and involved full-thickness scalp and cranium tissue (Figure 1).

**Figure 2:**
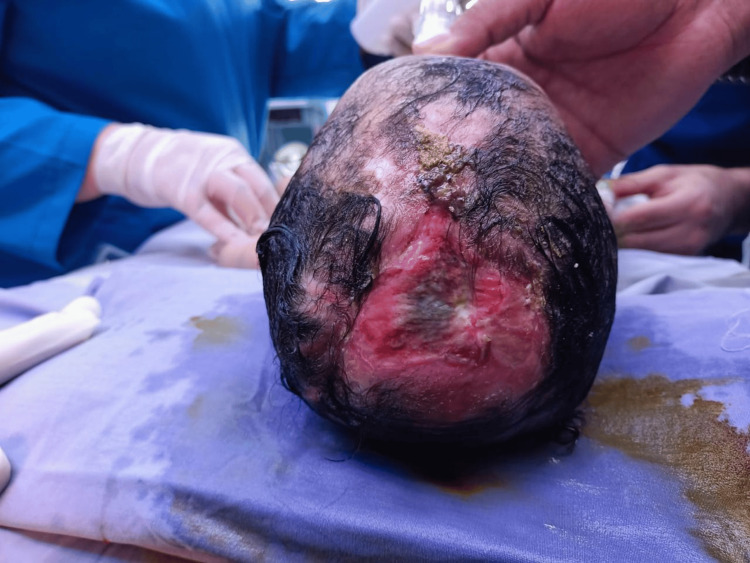
Scalp and cranium defect at two to three months old

Upon admission, the hemoglobin level was 9.4 mg/dL, which rapidly dropped to 7.8 mg/dL, following which he received a blood transfusion. Serum electrolytes and other labs were within normal limits. Throughout his hospitalization, the defect was complicated by a *Pseudomonas* wound infection, for which he received IV antibiotics. The patient underwent frequent dressing changes and wound inspections in the operating theater, with an STSG attempted at three months to reduce the defect size (Figure 2). However, the grafts were compromised due to recurrent infection.

The patient continued to receive IV antibiotics and underwent dressing changes until five months of age. At this point, while being hospitalized, a significant bleeding episode occurred, necessitating further surgical intervention for hemostasis and another attempt at skin grafting that failed once again.

**Figure 3:**
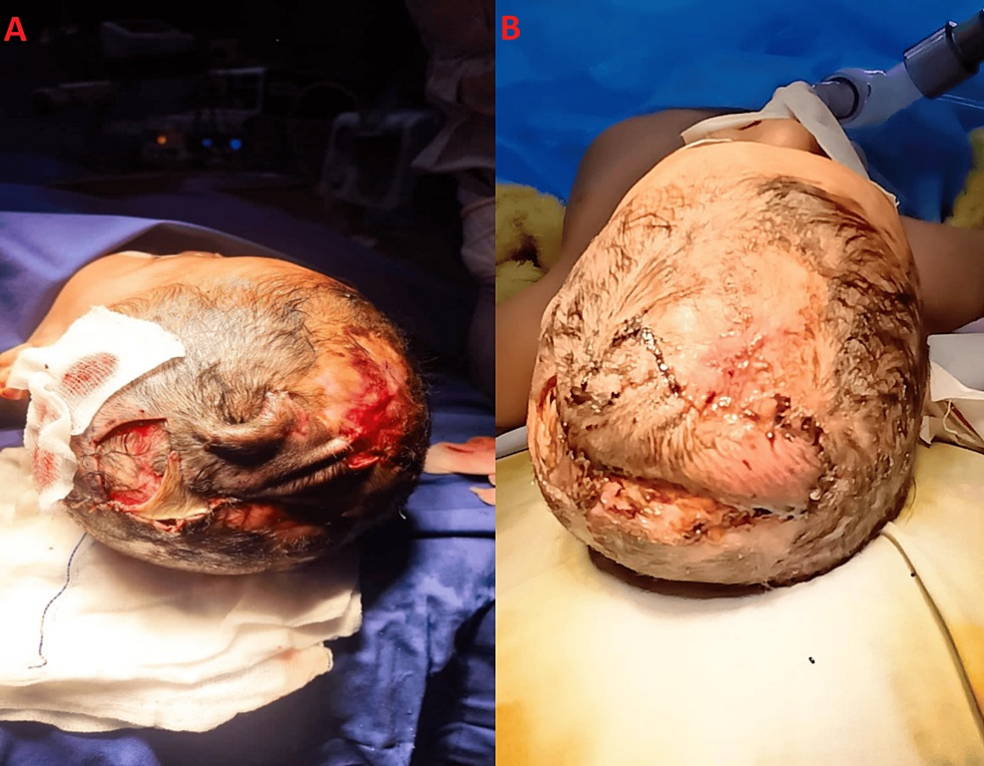
Frame A shows the transposition flap done at five months. Frame B captures the successful result of the transposition flap at the one-week follow-up.

The patient then underwent regular dressing changes twice a week, using various products to support healing. Another severe bleeding episode occurred when the patient was five months old and hospitalized, in which bipolar diathermy and pressure were applied in the theater to control the bleeding. During this procedure, a local transposition flap was performed over a normal hair-bearing skin bridge to address the defect. At the one-week follow-up, the flap was inspected; it showed excellent viability with 80% of the defect closed (Figure 3). Six weeks later, the flap was successfully divided, demonstrating favorable adherence over the raw area.

**Figure 4:**
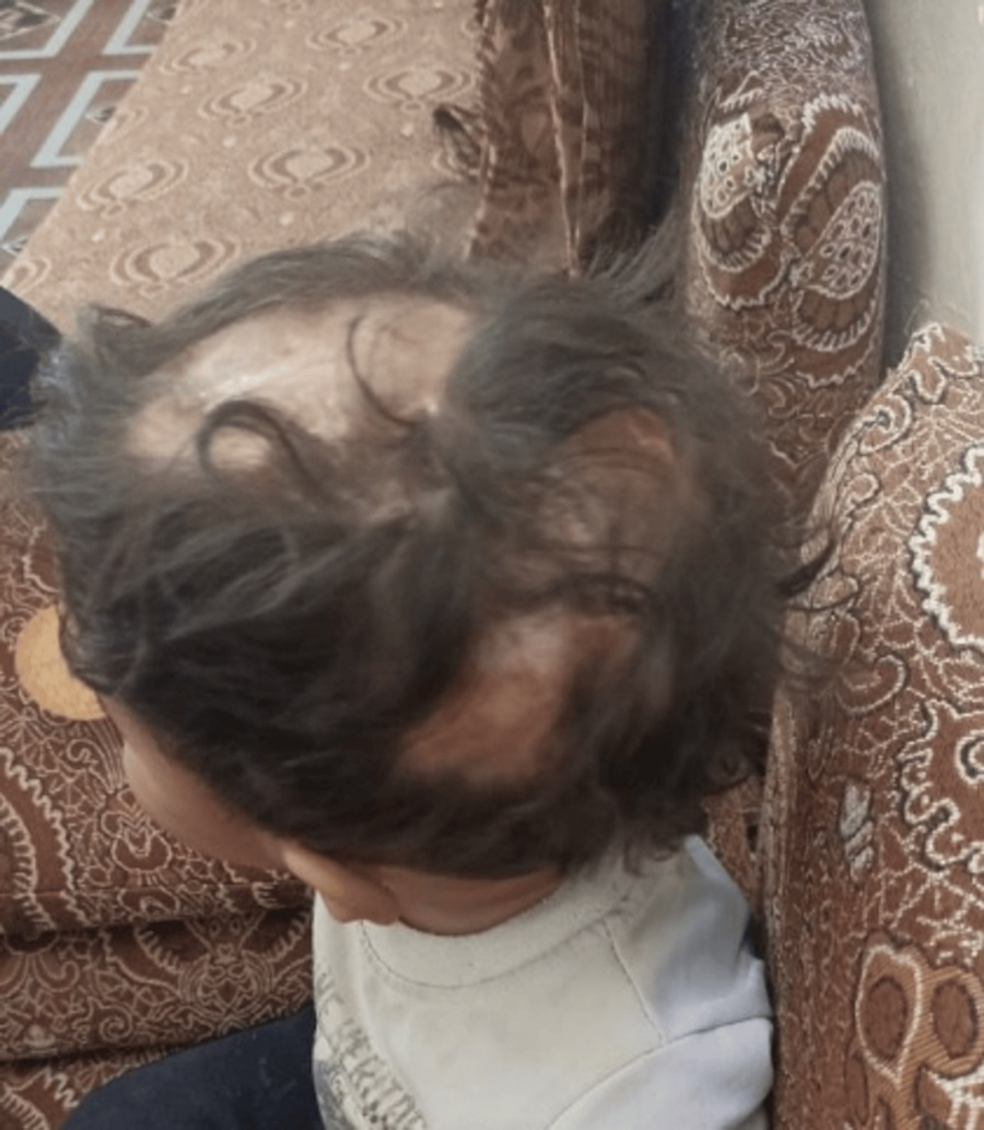
Image of the scalp taken at the follow-up after one year

The patient was discharged home by the age of six months. During follow-up, the patient showed positive progress with no recurrence of bleeding and an almost-good hair-bearing scalp. He is now a year and a half old, with normal development and growth, indicating favorable outcomes of the treatment approach implemented for his ACC (Figure 4).

## Discussion

Aplasia cutis congenita refers to the absence of skin at birth, a rare dermatological condition that typically occurs without any associated physical abnormalities. The causes of ACC are diverse, with multiple factors likely contributing to its onset [1]. It can manifest in any area of the body, and its clinical presentation and location may provide valuable clues to potential associated abnormalities [2]. Clinically, ACC exhibits significant variability in both expressivity and penetrance. Manifestations range from minor aesthetic anomalies to profoundly disabling defects, highlighting the diverse spectrum of presentations observed in affected individuals [8]. Table 1 shows the classification of ACC.

**Table 1:** Classification of ACC ACC: Aplasia cutis congenita, EB: Epidermolysis bullosa Reproduced from *Aplasia cutis congenita: A clinical review and proposal for classification* by Frieden IJ [7]. The table has been used with permission from Elsevier Inc.

Category	Body area affected	Associated abnormalities	Inheritance
Group 1			
Scalp ACC without multiple anomalies	Scalp, usually vertex	Cleft lip and palate; tracheoesophageal fistula; double cervix and uterus; patent ductus arteriosus; omphalocele; polycystic kidney; mental retardation; cutis marmoratatelangiectaticacongenita	Autosomal dominant or sporadic
Group 2			
Scalp ACC with associated limb abnormalities	Midline scalp	Limb reduction abnormalities; two to three syndactyly; clubfoot; nail absence or dystrophy; skin tags on toes; persistent cutis marmorata; encephalocele; woolly hair; hemangioma; heart disease; cryptorchidism; postaxial polydactyly (one family)	Autosomal dominant
Group 3			
Scalp ACC with associated epidermal and organoid nevi	Scalp; may be asymmetric	Corneal opacities; scleral dermoids; eyelid colobomas; psychomotor retardation; seizures	Sporadic
Group 4			
ACC overlying embryologic malformations	Abdomen, lumbar skin, scalp; any site	Meningomyeloceles; spinal dysraphia; cranial stenosis; congenital midline porencephaly; leptomeningeal angiomatosis; ectopia of ear; omphalocele; gastroschisis	Depends on underlying condition
Group 5			
ACC with associated fetus papyraceus or placental infarcts	Multiple, symmetric areas, often stellate or linear, on scalp, chest, flanks, axillae, and extremities	Single umbilical artery; developmental delay; spastic paralysis; nail dystrophy; clubbed hands and feet; amniotic bands	Sporadic
Group 6: ACC associated with EB			
Blistering, usually localized, without multiple congenital anomalies	Extremities	Blistering of skin and/or mucous membranes; absent or deformed nails; metatarsus varus; congenital absence of kidney (seen in cases of recessive, dystrophic EB; dominant, dystrophic EB; and EB simplex)	Depends on EB type: may be autosomal dominant or recessive
Widespread skin fragility with congenital anomalies	Large areas on extremities and torso	Pyloric or duodenal atresia; abnormal ears and nose; ureteral stenosis; renal abnormalities; arthrogryposis; amniotic bands; nail dystrophy	Autosomal recessive
Group 7			
ACC localized to extremities without blistering	Pretibial areas; dorsal aspects of hands and feet; extensor areas of wrists	None	Autosomal dominant or recessive
Group 8			
ACC caused by specific teratogens	Scalp (with methimazole); any area (with varicella and herpes simplex infections)	Imperforate anus (methimazole); signs of intrauterine infection with varicella and herpes simplex infections	Not inherited
Group 9			
ACC associated with malformation syndromes	Scalp; any location	Trisomy 13; 4p deletion syndrome; many ectodermal dysplasias; Johanson-Blizzard syndrome; focal dermal hypoplasia; amniotic band disruption complex; XY gonadal dysgenesis	Varies, depending on the specific syndrome

In this case report, we describe a newborn diagnosed with ACC affecting both the scalp and cranium, which are commonly affected areas. The patient initially presented with a life-threatening hemorrhage. It is noted in the literature that there is a potential risk of developing hemorrhages and meningitis in patients with ACC, although this complication is considered rare [9].

The etiology of ACC remains unclear, with various potential predisposing factors proposed in previous literature. These factors include maternal age, obstetric trauma, the number of pregnancies, and medications taken during pregnancy [9]. In the case presented, the infant is the first-born child of non-consanguineous parents and was delivered via cesarean section due to maternal pelvic inadequacy and postdate pregnancy, resulting in fetal distress. Additionally, the mother experienced transient polyhydramnios during the sixth month of pregnancy, which resolved after dietary adjustments. While these factors may have contributed to the development of ACC, definitive causal links cannot be established.

The management of ACC is complicated and is largely attributed to the unique presentation and heterogeneity observed in each case. Treatment strategies are personalized, considering variables such as the size and depth of the skin defect, the presence of any associated abnormalities, the risk of complications, and the expertise of the medical team. Options encompass a spectrum, from observation to surgical intervention or a blend of both modalities [2]. Collaboration with a multidisciplinary team is essential, typically including a pediatrician, pediatric dermatologist, pediatric plastic surgeon, and pediatric neurosurgeon, as necessary. This collaborative approach ensures comprehensive evaluation and optimal management of ACC cases.

In our case, the management took part in several stages. First, at the age of three months, an attempt was made to reduce the defect size with an STSG, but recurrent infections compromised the grafts, leading to ongoing management challenges. At the age of five months, the patient experienced severe bleeding, requiring surgical intervention and another failed skin graft attempt. Subsequently, regular dressing changes were initiated, and another bleeding episode prompted a surgical procedure with bipolar diathermy and pressure to control bleeding. This was followed by a successful local transposition flap surgery six weeks later, resulting in favorable healing.

A preventive method reported in the literature for managing bleeding from exposed dura involves early coverage of the scalp defect with full-thickness rotated flaps after birth, which is deemed the most reliable approach for preventing spontaneous hemorrhage and infection [10]. In our case, the patient presented early with hemorrhage, which was initially managed by applying direct pressure with gauze, followed by STSG when bleeding became recurrent.

Split-thickness skin grafting for primary scalp defects and donor site coverage post-scalp flap rotation has been documented [11,12], though challenging in neonates due to their thin skin. In emergent scenarios where full-thickness flaps fail [10], STSG can be considered as a last resort over exposed dura [13]. While split-thickness grafts may not support osseous regeneration over dura, they may be suitable for defects over intact bone and periosteum in older children [14]. In our case, we attempted the transposition flap due to recurrent infections compromising the grafts.

## Conclusions

The presentation of ACC varies from being asymptomatic to leading to life-threatening hemorrhage, as in our patient. Therefore, managing ACC requires a comprehensive approach involving a multidisciplinary team. Personalized treatment plans, including observation and surgical intervention using an STSG, are essential due to the wide spectrum of presentation and severity of cases. Thus, it is essential to approach each case of ACC individually, taking into account its unique associated factors and characteristics.

## References

[1] Humphrey SR, Hu X, Adamson K, Schaus A, Jensen JN, Drolet B (2018). A practical approach to the evaluation and treatment of an infant with aplasia cutis congenita. J Perinatol.

[2] Bharti G, Groves L, David LR, Sanger C, Argenta LC (2011). Aplasia cutis congenita: clinical management of a rare congenital anomaly. J Craniofac Surg.

[3] Coi A, Barisic I, Garne E (2023). Epidemiology of aplasia cutis congenita: a population-based study in Europe. J Eur Acad Dermatol Venereol.

[4] Martinez-Regueira S, Vazquez-Lopez ME, Somoza-Rubio C, Morales-Redondo R, Gonzalez-Gay MA (2006). Aplasia cutis congenita in a defined population from northwest Spain. Pediatr Dermatol.

[5] Sánchez-Pedreño Guillén P, Pichardo AR, Martínez FC (1985 ). Aplasia cutis congenita. J Am Acad Dermatol.

[6] Sagi EF, Linder N, Shouval D (1985). Papular acrodermatitis of childhood associated with hepatitis A virus infection. Pediatr Dermatol.

[7] Frieden IJ (1986 ). Aplasia cutis congenita: a clinical review and proposal for classification. J Am Acad Dermatol.

[8] Scott FP (1967). Congenital skin defects. Dermatologica.

[9] Deeken JH, Caplan RM (1970 ). Aplasia cutis congenita. Arch Dermatol.

[10] Conway H, Johnson G Jr (1956). Congenital absence of the scalp and skull. Ann Surg.

[11] Yang JY, Yang WG (2000). Large scalp and skull defect in aplasia cutis congenita. Br J Plast Surg.

[12] Perlyn CA, Schmelzer R, Govier D, Marsh JL (2005). Congenital scalp and calvarial deficiencies: principles for classification and surgical management. Plast Reconstr Surg.

[13] Kim CS, Tatum SA, Rodziewicz G (2001). Scalp aplasia cutis congenita presenting with sagittal sinus hemorrhage. Arch Otolaryngol Head Neck Surg.

[14] Bajpai M, Pal K (2003). Aplasia cutis cerebri with partial acrania — total reconstruction in a severe case and review of the literature. J Pediatr Surg.

